# Progression Free Survival, Overall Survival, and Relapse Rate in Endometrioid Ovarian Cancer and Synchronous Endometrial-Ovarian Endometrioid Cancer (SEO-EC): Results from a Large Retrospective Analysis

**DOI:** 10.3390/medicina58121706

**Published:** 2022-11-23

**Authors:** Basilio Pecorino, Antonio Simone Laganà, Vito Chiantera, Martina Ferrara, Andrea Benedetto Di Stefano, Mariano Catello Di Donna, Felice Sorrentino, Luigi Nappi, Mislav Mikuš, Paolo Scollo

**Affiliations:** 1 Maternal and Child Department, Obstetrics and Gynecology Cannizzaro Hospital, University of Enna “Kore”, 95126 Catania, Italy; 2 Unit of Gynecologic Oncology, ARNAS “Civico-Di Cristina-Benfratelli”, 90127 Palermo, Italy; 3 Department of Health Promotion, Mother and Child Care, Internal Medicine and Medical Specialties (PROMISE), University of Palermo, 90133 Palermo, Italy; 4 Department of Surgical, Oncological and Oral Sciences (Di. Chir. On. S.), University of Palermo, 90133 Palermo, Italy; 5 Department of Medical and Surgical Sciences, Institute of Obstetrics and Gynaecology, University of Foggia, 71121 Foggia, Italy; 6 Department of Obstetrics and Gynecology, University Hospital Centre Zagreb, 10 000 Zagreb, Croatia

**Keywords:** endometrioid ovarian cancer, synchronous endometrial-ovarian endometrioid cancer, lymphadenectomy, gynecological surgery, gynecological oncology, gynecological cancer

## Abstract

*Background and Objectives*: We aimed to evaluate Progression Free Survival (PFS), Overall Survival (OS), and relapse rate in women affected by endometrioid ovarian cancer and synchronous endometrial-ovarian endometrioid cancer (SEO-EC). As secondary outcome, we assessed whether systematic pelvic and para-aortic lymphadenectomy could be considered a determinant of relapse rate in this population. *Materials and Methods*: We performed a retrospective analysis of women with diagnosis of endometrioid ovarian cancer or SEO-EC between January 2010 to September 2020, and calculated PFS, OS and relapse rate. *Results*: In almost all the patients (97.6%) who underwent systematic pelvic and para-aortic lymphadenectomy, there were no lymph node metastases confirmed by histology. We did not find a significant difference (*p* = 0.6570) for the rate of relapse in the group of women who underwent systematic pelvic and para-aortic lymphadenectomy (4/42; 9.5%) compared with the group of women who did not undergo the same procedure (1/21; 4.8%). During a median follow-up was 23 months, both PFS and OS were excellent. *Conclusions*: Women affected by early-stage low-grade endometrioid cancer and SEO-EC without apparent lymph node involvement at pre-operative imaging showed a very low rate of lymph node metastasis and similar relapse rate with or without lymphadenectomy.

## 1. Introduction

Epithelial ovarian cancer represents about 30% of gynecologic malignancy and it is the fourth malignant neoplasm causing cancer death in women [1]. According to the most recent classification by the World Health Organization, ovarian carcinoma could be differentiated in five principal histotypes: high-grade serous carcinoma, low-grade serous carcinoma, mucinous carcinoma, endometrioid carcinoma and clear cell carcinoma. Four-marker immunohistochemical panel (WT1/p53/napsin A/PR) can distinguish the five principal histotypes with high accuracy, and additional immunohistochemical markers can be used depending on the diagnostic considerations [2]. In particular, endometrioid carcinomas are composed of the same molecular subtypes (POLE-mutated/mismatch repair-deficient/no specific molecular profile/p53-abnormal) with the same prognostic stratification as their endometrial counterparts [3].

Endometrioid ovarian cancer accounts about 10% of all epithelial tumors. In most cases, it develops in perimenopausal women and is diagnosed at early stage [4]. In approximately 42% of the cases, endometrioid ovarian cancer is associated with ovarian and/or pelvic endometriosis [5]. In particular, atypical endometriosis was found as the precursor of endometrioid ovarian cancer and clear cells cancer in 15–32% of the cases [6].

Overall, about 10% of women with ovarian cancer and 5% of women with endometrial cancer have synchronous endometrial-ovarian endometrioid cancer (SEO-EC). This population includes three main groups: endometrial cancer with adnexal (ovary and/or Fallopian tube) metastasis; ovarian cancer with endometrial metastasis; synchronous primary endometrial and ovarian cancer. A recent data analysis [7] suggested that SEO-EC and endometrioid ovarian cancer may represent independent primary tumors rather than metastatic disease. In particular, using high-depth targeted massively parallel sequencing of sporadic SEO-EC and endometrioid ovarian cancer, they were found to constitute independent cancers lacking somatic mutations in common.

Accumulating evidence suggests that women with SEO-EC have better prognosis than patients with primary ovarian or endometrial cancer with metastasis [8]. Nevertheless, available data about SEO-EC are still not robust to draw firm conclusion about the predictors of prognosis.

In this scenario, we aimed to evaluate Progression Free Survival (PFS), Overall Survival (OS), and relapse rate in women affected by endometrioid ovarian cancer and SEO-EC. As secondary outcome, we assessed whether systematic pelvic and para-aortic lymphadenectomy could be considered a determinant of relapse rate in this population.

## 2. Materials and Methods

We performed a retrospective analysis of women who underwent surgery for suspected ovarian cancer with incidental synchronous endometrial cancer, and included cases with final histological diagnosis of endometrioid ovarian cancer or SEO-EC, between January 2010 to September 2020, after approval by the independent Institutional Review Board of the Cannizzaro Hospital (ID: 09/2022). The design, analysis, interpretation of data, drafting, and revisions conform to the Helsinki Declaration, the Committee on Publication Ethics guidelines, and the REporting of studies Conducted using Observational Routinely-collected health Data (RECORD) Statement [9], available through the Enhancing the Quality and Transparency of Health Research Network. The data collected were anonymized, taking into account the observational nature of the study, without personal data that could lead to formal identification of the patient. The investigators did not perform any experimental intervention, and based the clinical management on the international guidelines available at the moment of the diagnosis/treatment. Each patient enrolled in this study signed informed consent to allow data collection and analysis for research purposes. The study was not advertised. No remuneration was offered to the patients to give consent to be enrolled in this study.

We collected data about age, Body Mass Index (BMI), number of pregnancies, previous endometriosis diagnosis, International Federation of Gynecology and Obstetrics (FIGO) stage according to the old classification [10] (differentiation level, unilateral or bilateral ovarian invasion, tubal invasion, peritoneal and/or lymph nodal metastases), types of surgical procedure (all the women underwent upfront surgery), eventual adjuvant therapy (chemotherapy, CHT, and/or radiotherapy, RT) which was decided in a multidisciplinary tumor board, and follow-up. Follow-up consisted of gynecologic visit, transvaginal ultrasound scan, chest and abdomen computed tomography (CT) and CA125 dosage, every 3 months in the first 2 years and every 6 months in the next 3 years.

PFS has been defined as the length of time from surgery to the 1st disease relapse or death from any cause. The OS has been defined as the length of time from as the time from treatment to death, regardless of disease recurrence. The survival curves have been estimated by Kaplan-Meier analysis. Comparison of the relapse rate between the group of women who underwent systematic pelvic and para-aortic lymphadenectomy and the group of women who did not undergo the same procedure was performed by Pearson’s chi-squared test.

## 3. Results

During the study period, 63 patients with endometrioid ovarian cancer or SEO-EC diagnosis underwent primary surgery: 36 (57.1%) patients had endometrioid ovarian cancer diagnosis and 27 (42.9%) patients had SEO-EC diagnosis.

Mean age in the overall population was 47 (range 21–77) years, mean BMI 28.7 (range 19–46), almost half (52.4%) had no pregnancies, and 7 (11%) had a history of endometriosis.

Regarding the different types of upfront surgery, 42 (66.8%) women underwent complete staging surgery with pubis-subxiphoid longitudinal laparotomy, hysterectomy and bilateral adnexectomy, infracolic and gastrocolic omentectomy, systematic pelvic and para-aortic lymphadenectomy; 8 (12.7%) underwent pubis-subxiphoid longitudinal laparotomy, hysterectomy with bilateral adnexectomy, infracolic and gastrocolic omentectomy and bulky lymph node excision; 6 (9.5%) women underwent pubis-subxiphoid longitudinal laparotomy, hysterectomy and bilateral adnexectomy, infracolic and gastrocolic omentectomy; 4 (6.3%) women underwent suprapubic transversal laparotomy with hysterectomy and bilateral adnexectomy (in those cases, incomplete surgery was done because the diagnosis was obtained only by definitive histology; intraoperative frozen sections were not required since malignancy was not suspected at the time of surgery); 3 (4.7%) underwent operative laparoscopy with unilateral adnexectomy (in those cases, patients refused cytoreductive surgery due to high anesthesiologic risk). The three women who underwent incomplete surgery for unsuspected ovarian cancer were all stage IA, endometrioid histotype, grading G1: in those cases, patients did not undergo surgical restaging due to high anesthesiologic risk.

Based on definitive histology, ovarian involvement was unilateral in 44 cases (70%), and bilateral in 19 cases (30%). In 60 (95.2%) cases, patients had no tubal involvement; in the remaining 3 (4.8%) cases with tubal involvement, two women had endometrioid ovarian cancer and one had SEO-EC. The mean number of retrieved lymph nodes in the patients who underwent systematic pelvic and para-aortic lymphadenectomy was 24, which was recently suggested as adequate for ovarian malignancies [11]. In almost all the patients (97.6%) who underwent systematic pelvic and para-aortic lymphadenectomy, there were no lymph node metastases confirmed by histology; the only case with obturator lymph node metastases was found in one patient with high-grade SEO-EC (stage IIIA-IA). In all the 8 patients who underwent bulky lymph nodes excision, the lymph node status was negative. One case in the SEO-EC group had metastases to the omentum (stage IIIB-IA). Staging and grading is reported in Table 1. The overall complication rate in patients who underwent systematic pelvic and para-aortic lymphadenectomy was 4.7% (two patients developed lymphedema), which is line with the literature for primary debulking surgery, based on a previous data analysis on a large cohort [12].

62 patients out of 63, after surgery, were treated with 1st line adjuvant CHT based on Carboplatin and Paclitaxel for 6 cycles. One endometrioid ovarian cancer patient was staged as FIGO IA grade 1 and, according to national guidelines by the Italian Association of Medical Oncology, underwent only follow-up because of several comorbidities which did not allow standard adjuvant CHT treatment.

Median follow-up was 23 months (1–77 months). During the follow-up, there were five relapses (7.9%), all in the SEO-EC group, as described in Table 2.

The two SEO-EC patients with early relapse (after 3 and 5 months, respectively) previously underwent complete staging surgery with systematic pelvic and para-aortic lymphadenectomy, and completed the six cycles of CHT with Carboplatin and Paclitaxel.

The 54 months-relapse case with isolated para-aortic lymph node involvement found at Positron Emission Tomography (PET) also underwent adjuvant RT (the multidisciplinary tumor board decided for adjuvant RT focused on this isolated metastasis instead of secondary cytoreduction, due to several comorbidities of the patient), and is disease-free at the ongoing follow-up.

All the patients who relapsed were treated with adjuvant CHT and are currently disease-free at the ongoing follow-up, but one. Indeed, the 21-months relapse caused by vaginal vault involvement, treated with adjuvant radiotherapy and follow-up, died of acute lymphoid leukemia after 48 months. Overall, only these two patients (the 54 months-relapse with isolated para-aortic lymph node involvement and the 21-months relapse with vaginal vault involvement) underwent adjuvant RT (3.2%).

We did not find a significant difference (*p* = 0.6570) for the rate of relapse in the group of women who underwent systematic pelvic and para-aortic lymphadenectomy (4/42; 9.5%) compared with the group of women who did not undergo the same procedure (1/21; 4.8%).

**Figure 1:**
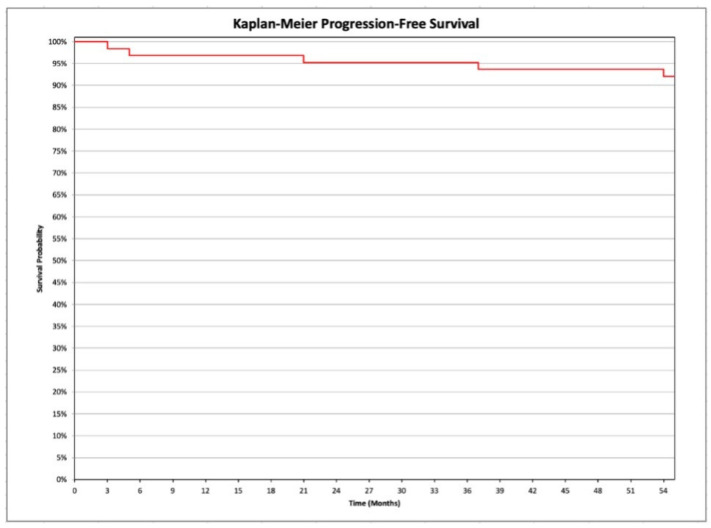
Kaplan-Meier analysis of the progression free survival in the investigated population (women affected by in endometrioid ovarian cancers, and by synchronous endometrial-ovarian endometrioid cancers).

**Figure 2:**
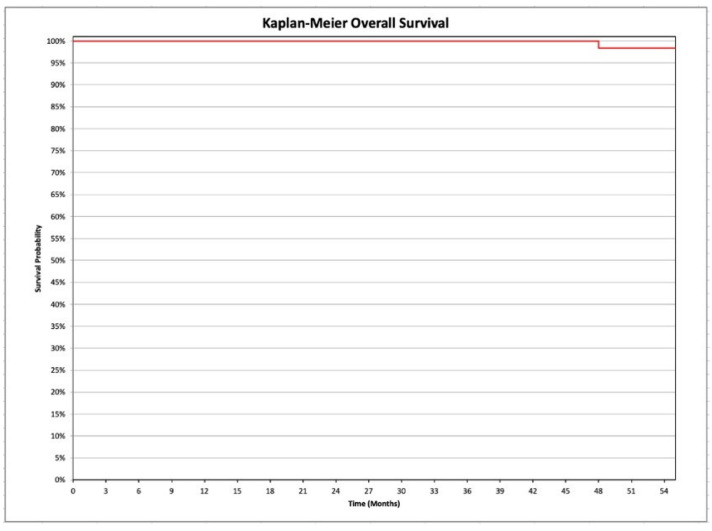
Kaplan-Meier analysis of the overall survival in the investigated population (women affected by in endometrioid ovarian cancers, and by synchronous endometrial-ovarian endometrioid cancers).

PFS analysis is reported in Figure 1, and OS in Figure 2. In particular, 98.4% of the population is still alive.

## 4. Discussion

The primary outcome of this retrospective analysis was aimed to evaluate PFS, OS, and relapse rate in women affected by endometrioid ovarian cancer and SEO-EC. As secondary outcome, we assessed the relapse rate in women who underwent systematic pelvic and para-aortic lymphadenectomy and women who did not undergo the same procedure. In our series, diagnosis and treatment was done at early stages in 97.6% of the cases, thus allowing good PFS and OS, confirming the endometrioid biological behavior [13]. In addition, we did not find significant differences (*p* = 0.6570) for the relapse rate, with or without systematic pelvic and para-aortic lymphadenectomy.

Women affected by ovarian and/or endometrial endometrioid cancer differ from any other ovarian/endometrial cancers with different histology [14]. Previous analysis about low-grade endometrioid carcinomas have found a clonal relationship between endometrial and ovarian carcinomas in the vast majority of cases [15], suggesting that this type of ovarian cancer may arise from the endometrial microenvironment and then extend to the ovary.

According to our data analysis, endometrioid ovarian cancer and SEO-EC are usually diagnosed at early stage, and therefore treatment is associated with good prognosis. Our study cohort has an average age of 47 years, which is in line with similar studies and accounts for the earlier onset compared with other types of ovarian cancer [16]. In addition, average BMI was 28.7, confirming a potential link between overweight/obesity and endometrioid cancer [17], probably due to the up-regulation of aromatase activity and the associated pro-estrogenic environment. Interestingly, a very low rate (*n* = 7; 11%) of women in our cohorts had a history of endometriosis, compared to literature data (42%) [5], although we cannot make a robust hypothesis about this discrepancy. In addition, 29% (18/63) of patients included in our analysis are affected by grade 3 tumor, despite SEO-EC is a relatively common scenario in clinical practice and the tumors at both sites are usually low-grade carcinomas.

According to recent evidence from microarray gene expression profiling, endometrioid ovarian cancer shows altered KRAS, BRAF, PIK3CA, CTNNB1, ARID1A and PPP2R1A expression, with a peculiar molecular profile different from high-grade serous cancers, which have a mesenchymal cell type, characterized by overexpression of N-cadherin and P-cadherin and low expression of differentiation markers, including CA125 and MUC1 [18].

The biological behavior also reflects the risk of lymph node metastasis: according to previous reports [19], lymph node metastasis incidence is 19% in serous high-grade, 2.7% in serous low-grade, 13% in endometrioid, 20.8% in clear cell, and 1.7% in mucinous carcinomas, respectively. In our series, we found lymph node metastasis in only one (1.5%) case (obturator lymph node metastasis in a case with high-grade SEO-EC, stage IIIA-IA), similar to the rate (2.1%) reported in another large analysis from the National Cancer Institute’s Surveillance, Epidemiology, and End Results database [19].

Overall, we did not find lymph node metastases confirmed by histology in almost all (97.6%) the patients who underwent systematic pelvic and para-aortic lymphadenectomy; in addition, we did not find significant differences (*p* = 0.6570) for the relapse rate with or without systematic pelvic and para-aortic lymphadenectomy. Based on similar results, other authors suggested to consider to avoid systematic lymph node dissection in patients affected by early-stage low-grade endometrioid cancer and SEO-EC without apparent lymph node involvement at pre-operative imaging [20]. Interestingly, we found higher rate of recurrence in the full staged patients (9.5% vs. 4.8%), despite it was not statistically significant. Systematic lymphadenectomy is indeed associated with high morbidity, even when performed by skilled surgeons [21,22] and, for this reason, the choice to perform this procedure should be carefully balanced and based on the available evidence. In this scenario, recent and promising data suggested that sentinel-node biopsy in early-stage ovarian cancer is feasible and has the potential to provide reliable and useful information on nodal status, and may allow the avoidance of systematic lymphadenectomy in the majority of patients [23,24].

Interestingly, all the recurrences were found in the SEO-OC group: from this perspective, it may be possible that this subgroup could be considered at higher risk of relapse due to a more aggressive behavior compared to endometrioid ovarian cancer. In particular, two patients diagnosed with stage I SEO-OC (IC-IA) experienced a very fast recurrence (3 and 5 months) despite an adjuvant treatment, despite they both underwent complete staging surgery with systematic pelvic and para-aortic lymphadenectomy, and completed the six cycles of CHT with Carboplatin and Paclitaxel.

Several limitations should be considered for a proper data interpretation: first of all, the retrospective nature of the data collection and analysis could be considered an intrinsic and significant bias; second, we used the old FIGO classification system [10], despite some patients were enrolled after the publication of the new classification in 2014 [8]; third, we had a very high rate of adjuvant treatment (98.4%) considering a 24.2% of grade 3 endometrioid ovarian cancer, despite guidelines by the European Society of Medical Oncology (ESMO) and the European Society of Gynecological Oncology (ESGO) does not recommend adjuvant treatment in case of IA G1-2 endometrioid ovarian cancer, and only optional adjuvant chemotherapy in case of full staged IC1 G1-2 patients [25]; fourth, the follow-up was short: indeed, a long-term survival analysis is needed to analyze survival data in early-stage endometrioid ovarian and SEO-OC; fifth, we did not compare the prognosis between of patients with endometrioid ovarian cancer and SEO-EC; finally we did not provide two distinct Kaplan-Meier curves showing the PFS and OS of patients with systematic lymphadenectomy and those without the procedure.

Based on our data analysis and literature data, we acknowledge the importance to integrate all available clinicopathologic, immunohistochemical and molecular data in the assessment of problematic diagnostic cases, such as the determination of tumor origin when there is synchronous, multifocal involvement of gynecologic tract sites, for example the endometrium and the ovary [26].

## 5. Conclusions

According to our data analysis, women affected by early-stage low-grade endometrioid cancer and SEO-EC without apparent lymph node involvement at pre-operative imaging showed a very low rate of lymph node metastasis and similar relapse rate with or without lymphadenectomy. Nevertheless, given the relatively low number of enrolled patients, the present analysis was underpowered and definitive conclusions cannot be drawn from these observations. Further research powered to detect a clinically significant association is needed to determine the most appropriate primary treatment for these women, including the value of systematic lymphadenectomy as part of cytoreductive surgery in this setting of patients.

## Figures and Tables

**Table 1:** Staging and grading in endometrioid ovarian cancer and synchronous endometrial-ovarian endometrioid cancer populations.

	Endometrioid Ovarian Cancer, *n* (%)		SEO-EC ^1^, *n* (%)	
FIGO ^2^ Stage	IA	8 (22.2%)	IA-IA ^3^	3 (11.1%)
	IC	20 (55.6 %)	IA-IB ^3^	1 (3.7%)
	IIA	1 (8.8%)	IB-IA ^3^	3 (11.1%)
	IIB	3 (8.3%)	IC-IA ^3^	15 (55.5%)
	IIC	4 (11.1%)	IC-IIIA ^3^	1 (3.7%)
			IIB-IA ^3^	1 (3.7%)
			IIC-IIIA ^3^	1 (3,7%)
			IIIA-IA ^3^	1 (3,7%)
			IIIB-IA ^3^	1 (3,7%)
Grade	G1	3 (9.1 %)	G1 ^4^	2 (6.7%)
	G2	22 (66.7%)	G2 ^4^	18 (60%)
	G3	8 (24.2%)	G3 ^4^	10 (33.3%)

^1^ SEO-EC: synchronous endometrial-ovarian endometrioid cancer. ^2^ FIGO: International Federation of Gynecology and Obstetrics (old classification [10]). ^3^ The first staging information refers to ovarian cancer, the second one refers to endometrial cancer (for instance, IIB-IA means that the staging is IIB for ovarian cancer and IA for endometrial cancer). ^4^ Grading in SEO-EC groups refers to endometrial cancer.

**Table 2:** Relapses synchronous endometrial-ovarian endometrioid cancer populations.

	FIGO ^1^ Stage	Time from Surgery	Site of Relapse
Synchronous endometrial-ovarian endometrioid cancer	IC ovary/IA endometrium	3 months	Groin-femoral lymph nodes
	IC ovary/IA endometrium	5 months	Pelvic lymph nodes
	IIC ovary/IA endometrium	21 months	Vaginal vault
	IC ovary/IA endometrium	37 months	Pelvic lymph nodes
	IIIA ovary/IA endometrium	54 months	Para-aortic lymph node

^1^ FIGO: International Federation of Gynecology and Obstetrics (old classification [10]).

## Data Availability

Full anonymized dataset will be available from the first author (B.P.) on reasonable request.
